# Author Response: Letter to the Editor Regarding “Intraretinal Hyper-Reflective Foci Are Almost Universally Present and Co-Localize With Intraretinal Fluid in Diabetic Macular Edema”

**DOI:** 10.1167/iovs.66.11.36

**Published:** 2025-08-14

**Authors:** Esther L. von Schulthess, Andreas Maunz, Usha Chakravarthy, Nancy Holekamp, Daniel Pauleikhoff, Katie Patel, Isabel Bachmeier, Siqing Yu, Yaniv Cohen, Mahnaz Parian-Scherb, Ian L. Jones, Kara Gibson, Jeffrey R. Willis, Carl Glittenberg, Rishi P. Singh, Sascha Fauser

**Affiliations:** 1 Roche Pharma Research and Early Development, Roche Innovation Center Basel, F. Hoffmann-La Roche Ltd., Basel, Switzerland; 2 Queen's University of Belfast, Royal Victoria Hospital, Belfast, United Kingdom; 3 Pepose Vision Institute, Chesterfield, Missouri, United States; 4 Augenzentrum am St. Franziskus Hospital, Münster, Germany; 5 Roche Products Ltd., Welwyn Garden City, United Kingdom; 6 Genentech, Inc., South San Francisco, California, United States; 7 Center for Ophthalmic Bioinformatics, Cole Eye Institute, Cleveland Clinic, Cleveland, Ohio, United States. E-mail: esther.von_schulthess@roche.com.

##  

We appreciate the thoughtful comments provided by Midena and Frizziero^1^ regarding our publication “Intraretinal Hyper-Reflective Foci Are Almost Universally Present and Co-Localize With Intraretinal Fluid in Diabetic Macular Edema.”^2^ Their insights into the importance of image quality, the definition of hyperreflective foci (HRF) subtypes, and the implications of HRF characteristics in the context of artificial intelligence (AI) analysis are highly valuable.

We acknowledge the role of image acquisition parameters, such as the SPECTRALIS Automatic Real Time (ART) function, in ensuring accurate and reproducible biomedical image segmentation, especially for small objects such as HRF. Our study employed standardized spectral-domain optical coherence tomography (SD-OCT) imaging protocols to minimize variability, but we recognize that higher ART thresholds and high-resolution (HR) scans could further enhance detection of smaller HRF (<30 µm). We agree that this is important and note that the scans used for this work had a quite narrow range of ART values, between 9 and 12, and that a study by the Medical University of Vienna suggested 16 as optimal for segmentations^3^ (too high ART values result in loss of detail due to averaging). Future work could explore the impact of such refinements on AI-based HRF classification.

Robust segmentation presents the first important step in further exploring the role of HRF and the differentiation of HRF subtypes, but the question of etiology (inflammatory cells vs. lipid deposits) has not yet been fully addressed. Our study focused on objects between 20 and 50 µm in diameter, displaying reflectivity similar to the retinal pigment epithelium (RPE). We recognize the importance of refining automated classification models to differentiate among HRF of varied origins and did not account for further properties, such as clustering or backshadowing. However, the segmentation model does exclude blood (hemorrhage) and other material, as per the grading guidelines and our internal expert panel review of the trained model. Additionally, our annotations were limited to a 20-µm minimal size due to the limitation of the transversal resolution of SD-OCT, which is around 15 µm/pixel in standard mode, in which our scans were taken.

We also appreciate the discussion by Midena and Frizziero^1^ on the implications of HRF quantification methods. HRF volume estimation is indeed an effective approach for assessing exudative changes but may be less suited for evaluating cellular clusters such as microglia. Future studies could consider alternative quantification techniques, such as direct cell aggregate counting or spatial distribution analysis, to better capture neuroinflammatory processes in diabetic retinopathy.

In conclusion, we value the constructive feedback provided by Midena and Frizziero and recognize the need for continued refinement in HRF characterization and AI-based analysis. We hope our study contributes to advancing standardized HRF assessment and lays the groundwork for further investigation into the diverse biological implications of HRF in diabetic macular edema.
